# High-Throughput RNA Sequencing of Mosaic Infected and Non-Infected Apple (*Malus × domestica* Borkh.) Cultivars: From Detection to the Reconstruction of Whole Genome of Viruses and Viroid

**DOI:** 10.3390/plants11050675

**Published:** 2022-03-01

**Authors:** Sajad U. Nabi, Virendra K. Baranwal, Govind P. Rao, Sheikh Mansoor, Carmen Vladulescu, Wasim H. Raja, Basit L. Jan, Saleh Alansi

**Affiliations:** 1 ICAR-Central Institute of Temperate Horticulture, Srinagar 191132, India; sajad_patho@rediffmail.com (S.U.N.); wasimiari@gmail.com (W.H.R.); 2 Advanced Center for Plant Virology, Division of Plant Pathology, ICAR-Indian Agricultural Research Institute, New Delhi 110012, India; gpraossrp@gmail.com; 3 Division of Biochemistry, FBSc, Sher-e-Kashmir University of Agricultural Science and Technology, Jammu 180009, India; 4 Department of Biology and Environmental Engineering, University of Craiova, 13, A.I.Cuza, 200585 Craiova, Romania; carmen_vldl@yahoo.com; 5 Department of Clinical Pharmacy, College of Pharmacy, King Saud University, Riyadh 11451, Saudi Arabia; basitlatief@gmail.com; 6 Botany and Microbiology Department, College of Science, King Saud University, Riyadh 11451, Saudi Arabia; alansi1975@yahoo.com

**Keywords:** next-generation sequencing, RNA-Seq, apple, Illumina, mosaic

## Abstract

Many viruses have been found associated with apple mosaic disease in different parts of the world. In order to reveal and characterize the viruses and viroids in symptomatic apple plants, next-generation sequencing (RNA seq.) of rRNA-depleted total RNA using Illumina Hiseq2500 was applied to two cultivars, Oregon Spur and Golden Delicious, with symptoms of mosaic and necrosis and one cultivar, Red Fuji, which was asymptomatic. The RNA sequencing detected five viruses, viz., apple necrotic mosaic virus (ApNMV), apple mosaic virus (ApMV), apple stem grooving virus (ASGV) and apple stem pitting virus (ASPV), apple chlorotic leaf spot virus (ACLSV), and one viroid i.e., apple hammerhead viroid (AHVd). RT-PCR amplification and sequencing also confirmed the presence of all these five viruses and viroids detected in HTS of total RNA. The complete genomes of five viruses and AHVd were reconstructed. The phylogenetic analysis of these viruses and AHVd revealed genetic diversity by forming subclusters with isolates from other countries. Recombination events were observed in all five viruses while single-nucleotide variants were detected only in ApMV and ApNMV. The absence of ApMV and ApNMV in asymptomatic samples from the same cultivars in an RT-PCR assay indicated that these two viruses are associated with mosaic disease of apples in India. This is the first viral genome analysis of symptomatic and asymptomatic apple plants and the first report of genome characterization of viruses associated with apple mosaic disease from India. High-throughput RNA sequencing is a powerful tool to characterize the genome of viruses and viroids in plants previously undetected by conventional methods. This would also help in the indexing and certification of large-scale germplasm.

## 1. Introduction

Apple (*Malus × domestica* Borkh.) belongs to the *Rosaceae* family and is one of the most important remunerative pome fruits grown across temperate regions of the world [1]. In India, the apple is cultivated mostly in northwestern Himalayan states viz., Jammu & Kashmir (J&K), Himachal Pradesh (HP) and Uttarakhand, and forms the backbone of their economy [2]. The productivity of apples in India (8 t/ha) is much lower as compared to the world average (17t/ha) and is severely affected by various diseases incited by bacteria, fungi, phytoplasmas and viruses [3,4]. Apple mosaic disease (AMD) is the most economically important viral disease, with widespread distribution and is a major threat to the apple industry throughout the world [5,6]). In infected leaves, AMD reduces the photosynthetic rate by 3–46%, which results in yield losses of 30–40% [7,8]. In the early 1930s, the apple mosaic virus (ApMV) was found to be associated with AMD. However, several other viruses, viz., apple necrotic mosaic virus (ApNMV), prunus necrotic ring spot virus (PNRSV) and cucumber mosaic virus (CMV), [9,10,11], have also been found associated with AMD. Based on immunological and molecular methods (DAS-ELISA and RT-PCR), ApMV was the only causal agent found to be associated with AMD from the states of J&K and HP in India up until 2019 [12,13,14]. However, in 2020, Nabi et al. reported the association of both ApMV and ApNMV with mosaic disease in several apple cultivars from India. As traditional methods such as ELISA, molecular hybridization and RT-PCR only detect targeted known viruses, it was not possible to detect non-targeted viruses or unknown viruses. This limitation of immuno-molecular methods has been overcome by high-throughput sequencing (HTS) technology. HTS has been used to unravel the virome in apple trees with different disease phenotypes, including mosaic, in various countries such as China, Korea and Japan [11,15]. As no virome analysis has been conducted in apple cultivars commercially grown in Indian conditions, a study was undertaken to characterize viruses/viroids associated with mosaic and necrotic symptoms in two cultivars of apples.

## 2. Results

### 2.1. Symptomatology and Incidence

**Figure 1:**
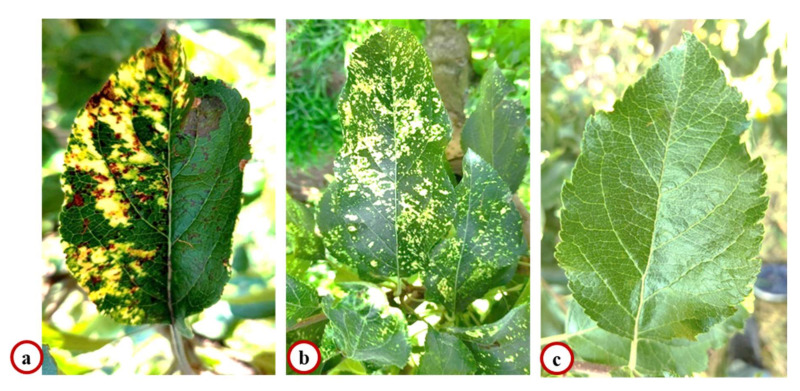
Symptoms of mosaic and necrosis on cultivar Oregon Spur (**a**); symptoms of mosaic randomly distributed on cultivar Golden Delicious (**b**); and asymptomatic cultivar Red Fuji bearing no symptom of either mosaic or necrosis (**c**).

A survey conducted in the field gene bank of ICAR-Central Institute of Temperate Horticulture showed the symptoms of mosaic scattered throughout the entire leaf lamina, ranging from pale yellow spots to large, contiguous, chlorotic spots along with necrosis on the leaves of apple tree cv. Oregon Spur (OS) (Figure 1a), while only mosaic symptoms were observed in the leaves of apple tree cv. Golden Delicious (GD) (Figure 1b). No symptoms were observed on the cultivar Red Fuji (RF) (Figure 1c). An incidence of approximately 30% and 5.5% of mosaic and necrosis-mosaic was observed on the cultivars GD and OS, respectively. 

### 2.2. Illumina HiSeq Sequencing Statistics

**Figure 2:**
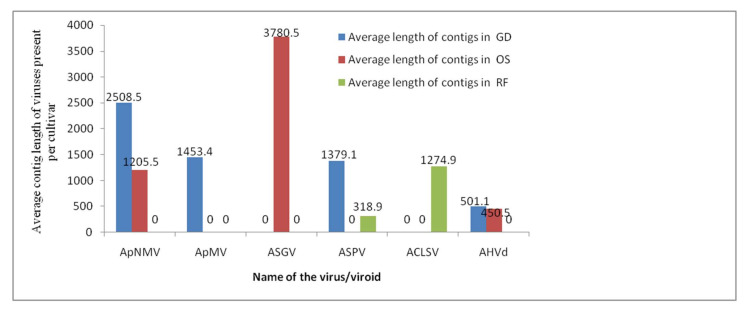
Average length of contigs obtained from de novo assembly using assemblers for ApNMV, ApMV, ASGV, ASPV, ACLSV and AHVd present per cultivar.

Illumina sequencing was performed on the total RNA isolated from the leaves of two apple cultivars for comparative Virome analyses. The raw reads obtained from Illumina Hiseq sequencing of the two cDNA libraries of GD, OS, and RF were further trimmed to remove adapters and low-quality reads to obtain clean reads of 120nts (Table 1). The sequence data were run through a classification tool, Kaiju (1.6.3 version), using the “NCBI BLAST nr+euk” database as a reference to identify the sequences of non-host origin. The library for the virus/viroid was constructed from a reference database available from NCBI, which was further used for the mapping of the reads. The total number of virus and viroid reads obtained from samples of OS, GD and RF are shown in Table 1. The de novo assembly and BLASTn searches resulted in assemblies of 97 virus/viroid-related contigs, with lengths ranging from 501–3780nt, as shown in Figure 2. The percentage distribution of reads pertaining to virus/viroid-associated reads per sample ranged from 0.05% to 1.36% of the clean reads for three samples. The samples of OS showed a higher number of virus/viroid-related reads, with 1.36% for viruses and 0.85 % for viroids from the clean reads, as shown in Table 1.

### 2.3. Viruses Detected in the Leaf Samples of Symptomatic and Asymptomatic Apple Cultivars

The virus and viroid communities differed among the two apple cultivars showing mosaic and necrotic symptoms. Viral/viroidal contigs from the OS sample (mosaic and necrosis) showed similarity with one viroid and three viral species. The viroid was identified as AHVd in the genus *Pelamoviroid* (family—*Avsunviroidae*). The viruses detected were ApNMV in the genus *Ilarvirus,* (family—*Bromoviridae*) and ASGV and ASPV in genera *Capillovirus* and *Foveavirus,* respectively, (family—*Betaflexiviridae*). The GD sample (mosaic) also exhibited the presence of AHVd and three viruses. However, the virus spectrum included ApNMV and ApMV in the genus *Ilarvirus*, (family—*Bromoviridae*) and ASGV in the genus *Capillovirus* (family—*Betaflexiviridae*). ApMV was associated with the only mosaic-showing cultivar (GD) whereas ApNMV was present in the cultivar showing both mosaic and mosaic–necrotic symptoms (OS). Moreover, ASGV was present in both apple cultivars but ASPV was absent in the GD cultivar. The asymptomatic apple cultivar RF was also found to be infected with two latent viruses, which included ACLSV in the genus *Trichovirus* and ASPV in the genus *Foveavirus* (family—*Betaflexiviridae*).

### 2.4. Distribution of Individual Virus/Viroid Reads

The results revealed that ApMV (63.2%), ApNMV (59.9%) and ACLSV (2.49%) were dominant in the total virus-related reads in the GD, OS and RF samples, respectively. Similarly, the viroid-associated reads revealed that the viroid reads were more abundant in the GD sample. The percentage of individual virus/viroid-associated reads present in each sample and the number of viral contigs for each individual virus in the cultivars OS, GD and RF obtained after de novo assembly ranged from 3 to 32, as presented in Table 2. The percentage of virus-associated reads revealed that the GD and OS samples collected from mosaic-infected trees had a higher number of sequences reads of the viruses ApMV and ApNMV, respectively.

### 2.5. Viruses/Viroid Confirmation by RT-PCR

**Figure 3:**
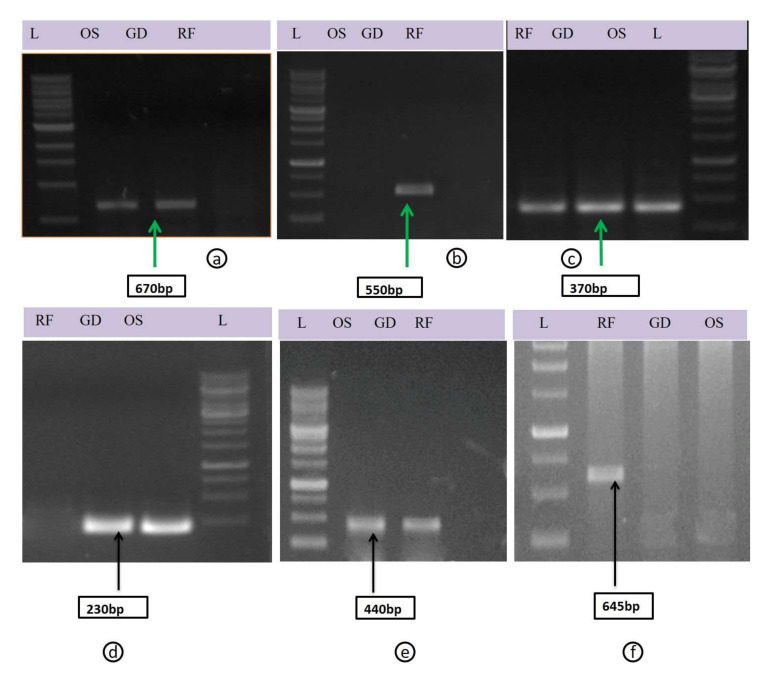
The PCR products from amplified region of viruses (**a**) ApNMV, (**b**) ApMV, (**c**) ASPV, (**d**) ASGV, (**e**) AHVd, (**f**) ACLSV L-1kb Ladder, OS: Oregon spur, GD: Golden Delicious, RF: Red Fuji.

To confirm the presence of viruses/viroids detected by HTS in two apple cultivars, RT-PCR was performed using specific primer pairs for viruses/viroids individually. The RT-PCR amplicons of 680 bp, 550 bp, 350 bp, 230 bp, 440 bp and 645bp had the expected sizes for ApNMV, ApMV, ASPV, ASGV, AHVd and ACLSV, respectively. Sequence analysis showed that the leaf samples were positive for viruses identified by RNA seq. The sequence results for the five viruses and the viroid showed 99–100% similarity to the virus/viroid detected by RNAseq, indicating that these viruses were actually present in total RNA isolated from apple trees (Figure 3a–e). 

### 2.6. Genome Reconstruction and Organization

**Figure 4:**
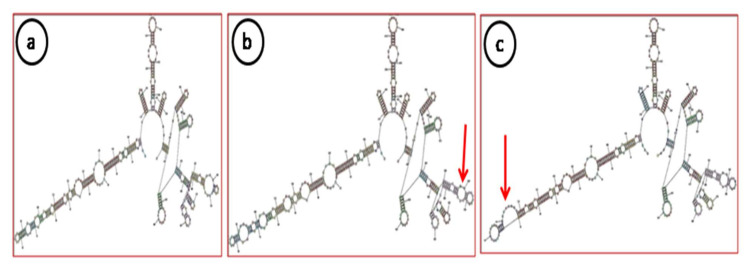
Secondary structure of apple hammerhead viroid: (**a**) Reference, (**b**) AHVd (GD), (**c**) AHVd (OS). The red arrows indicate the variations of nucleotides in our isolates in comparison to reference (MH043720).

The BLASTn search of contigs derived from each virus/viroid (ApMV, ApNMV, ASPV, ASGV and AHVd) was performed against a reference plant virus database. The position and sequence similarity of each contig associated with each virus was identified in the reference genome. The total sequence length retrieved for each identified virus is shown in Table 2. The complete genome of three viruses (ApMV-9kb, ASGV-7.6kb, ASPV-7kb and ACLSV-7.55kb) and one viroid (AHVd-434bp) was retrieved completely; however, for ApNMV, complete RNA1 (3.38kb), RNA 3 (1.96kb) and partial RNA2 (2.3kb) were retrieved from OS. The remaining 500bp of RNA 2 was amplified by designing primers as shown in Table S1 using RT-PCR, which was subsequently sequenced. The open reading frames (ORFs) of all five viruses were predicted using the NCBI-ORF Finder (Figure S1), and the genome organization was almost identical to reference strains. The whole genome of AHVd was also reconstructed from two samples (OS and GD). The secondary structure predicted using the RNA structure of both the isolates was similar to the reference strain (MH043720). The resulting conformations consisted of a rod-like domain containing the nucleotides forming a hammerhead structure. This was common to both variants and contained nine small stem-loops over the ribozyme head, which were almost preserved in both the variants with little variation in one of the stem loops at the nucleotide positions indicated in Figure 4a. The small nucleotide changes were also observed in the stem of AHVd-OS compared to AHVd-GD and the reference isolate as indicated in Figure 4b. The nucleotide changes may be due to co-variations or conversions of canonical base pairs into wobble base pairs. The genome sequences of each virus and viroid were submitted to GenBank and accession numbers were received. The details of the five viruses and one viroid along with their ORFs, sequence similarities, untranslated regions and accession numbers are presented in Table 3. 

### 2.7. Phylogenetic Analysis

**Figure 5:**
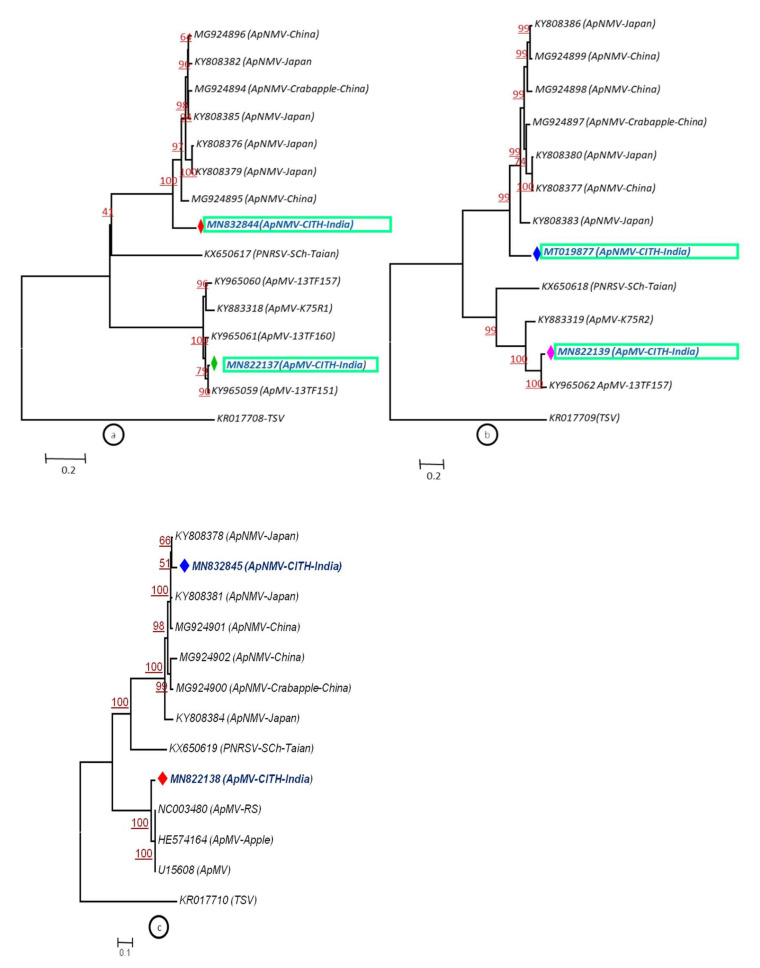
(**a**–**c**) Phylogenetic relationship of apple mosaic virus and apple necrotic mosaic virus using RNA 1 (**a**), RNA 2 (**b**) and RNA 3 (**c**) nucleotide sequence alignment through NJM at 1000 replications for each bootstrap value using MEGA 7.0; our ApNMV from cv.OS and ApMV from cv.GD isolates are highlighted with different colors in phylogeny. TSV (Tobacco streak virus) represents out-group in the same genus.

**Figure 6:**
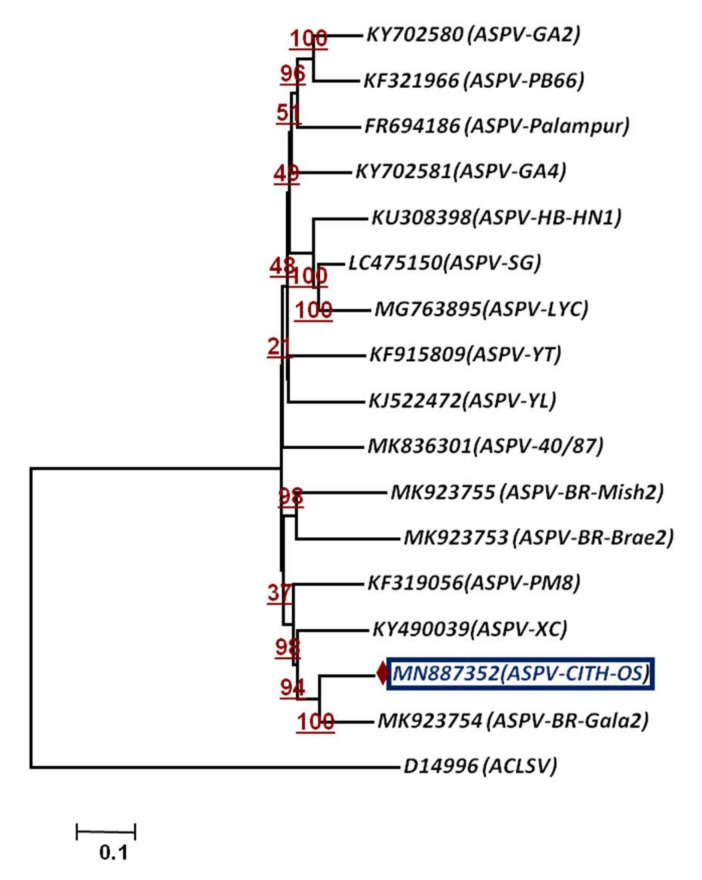
Phylogenetic relationship of apple stem pitting virus based on whole-genome sequence alignment through NJM at 1000 replications for each bootstrap value using MEGA 7.0; our ASPV isolate is highlighted with color in phylogeny. Apple chlorotic leaf spot virus (ACLSV) represents out-group.

**Figure 7:**
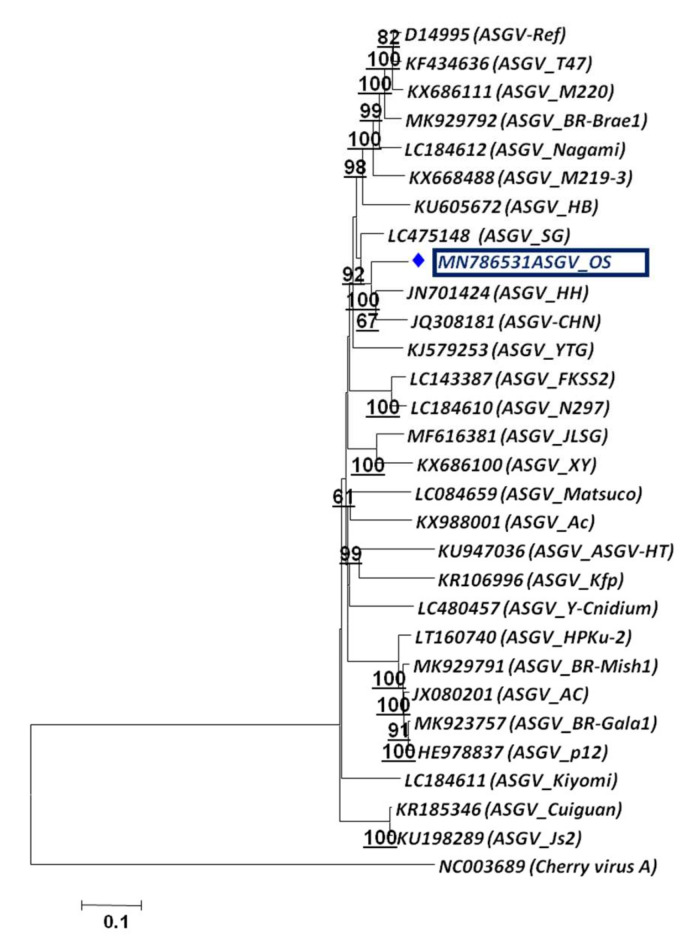
Phylogenetic relationship of apple stem grooving virus based on whole-genome sequence alignment through NJM at 1000 replications for each bootstrap value using MEGA 7.0; our ASGV isolate is highlighted with color in phylogeny. Cherry virus-A (CVA) represents out-group.

**Figure 8:**
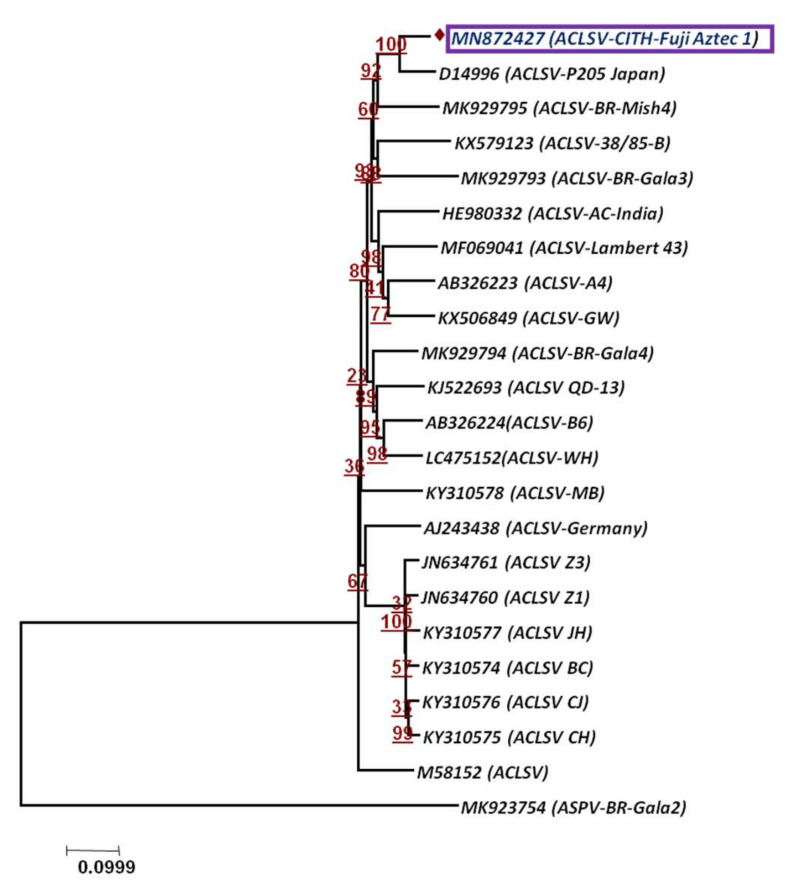
Phylogenetic relationship of Apple chlorotic leaf spot virus based on whole-genome sequence alignment through NJM at 1000 replications for each bootstrap value using MEGA 7.0; our ACLSV isolate is highlighted with color in phylogeny. Apple stem pitting virus (ASPV) represents out-group.

**Figure 9:**
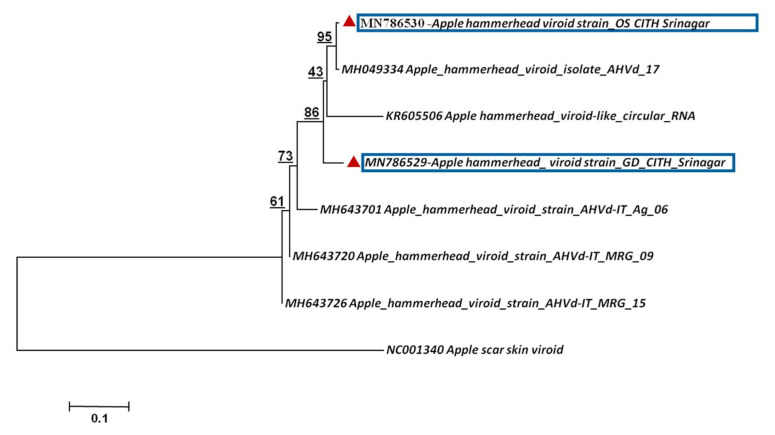
Phylogenetic relationship of apple hammerhead viroid based on whole-genome sequence alignment through NJM at 1000 replications for each bootstrap value using MEGA 7.0; our AHVd isolates are highlighted with color in phylogeny. The apple scar skin viroid (ASSVd) represents out-group.

To assess the phylogenetic relationship and taxonomic position of the identified viruses/viroid in the GD and OS apple tree samples, phylogenetic analysis was carried out based on the whole genome using the Neighbor-Joining method. The phylogenetic analysis was carried out individually based on RNA 1, RNA2 and RNA3 (Figure 5a–c) for both viruses, ApMV and ApNMV. The phylogenetic analysis revealed the formation of two separate clusters in all three phylogenetic trees. The ApMV (MN822137, MN822138 and MN822139) isolates in this study were found to be more closely related to American (KY965061 and KY883319) and Japanese isolates (KY965060 and KX650618). The ApNMV (MN832844, MN019877 and MN832845) isolate was more closely related to Chinese (MG924895) and Japanese (KY808376) isolates, as it clustered with these isolates in a single cluster. The ASPV (MN887352) was also found to be more closely related to Japanese isolates (MK923754) (Figure 6). The whole genome-based phylogenetic analysis clustered isolates in a single cluster with small subclusters, indicating the existence of higher variability among the isolates. The ASGV (MN786531) isolate was found to be more closely related to Japanese (JQ308181) and Brazilian isolates (JN701424) (Figure 7). The phylogenetic analysis of ACLSV (MN872427) revealed that this isolate was more closely related to the isolate from Japan (D14996) (Figure 8), and the presence of small subclusters indicates the presence of diversity among the isolates. Among the two viroid isolates, AHVd-GD (MN786529) was found to be closely related to the New Zealand isolate (KR605506) and AHVd-OS (MN786530) related to the Italian isolate (MH049334). From phylogenetic analysis and sequence similarity, the presence of intra-isolate variation is evident as both the isolates of AHVd were clustered in separate subclades with isolates from different countries, as shown in Figure 9, with a difference of approximately6% genome similarity.

### 2.8. Recombination and Single Nucleotide Variance (SNV) Analysis

The RDP4 program was used for the detection of recombination events with nine different algorithms in aligned genomes from identified viruses, and only recombination events supported by at least four algorithms were selected. Two recombination events were detected in RNA 2 of ApMV, and one event was detected in RNA3 of ApNMV. Two recombination events were detected in ACLSV, ASPV and ASGV each. The recombination events detected by the RDP4 program, along with major and minor parents and the *p*-value, are shown in Table 4 and Figure S2. The SNVs were present only in ApMV (1028) and ApNMV (350). 

## 3. Discussion

The word “Virome”, derived from “virus” and “genome”, is defined as all virus- or viroid-related genomes present in a specific tissue, organism or environment [16]. The virome study not only detects the viruses/viroids but also unravels the genome’s complexity, replication, mutation, recombination and nucleotide variations in virus/viroid genomes in certain situations [17]. Recent advances in the form of HTS technologies are powerful tools to detect and characterize untargeted viral pathogens with known/unknown genomic sequences. These technologies have found increased applications in unraveling the phytovirome, which contribute to the disease phenotype of a host plant [18]. In fact, most perennial fruit plants, including apples, are being vegetatively propagated and are considered as storehouses of a large number of viruses/viroids [19]. These technologies have opened new vistas not only for the detection and identification of known viruses, but also novel, newly emerging, unnoticed, un-described and divergent viruses/viroids to date [20]. The approach is also very helpful in the identification of viral co-infection in many plants [21,22]. Moreover, the increasing reports of association of ApNMV with mosaic from various countries such as China, Korea, Japan and India have changed the etiology of AMD, hence the need of the hour is to establish the etiology of mosaic disease using high-throughput methods in a large number of samples from various cultivars using HTS, which then should be validated via the development of infectious clones. The present study was undertaken to determine the phytovirome associated with mosaic disease and characterizes their whole genome using the RNA seq approach coupled with RT-PCR and Sanger Sequencing. We consider this to be the first application of HTS technology in assessing viruses/viriods associated with AMD infecting apple fruit crops in India. In the present investigation, two individual trees of apple cultivars were subjected to HTS to study the co-infecting virus/viroid populations. The two cultivars (GD and OS) were associated with either mosaic or necrosis or only mosaic. The sample collection was extracted from both symptomatic and asymptomatic apple cultivars grown in the same block with the same environmental conditions and cultivation practices, for comparative Virome analysis. The RNAseq-generated contigs were related to five viruses (ApMV, ApNMV, ASGV, ASPV and ACLSV) from four genera and one viroid, AHVd, belonging to the genus Pelamovioid. The presence of these five viruses and the viroid was confirmed in the two cultivars using RT-PCR and Sanger sequencing. Through HTS (Illumina platform), apple green crinkle disease was found to be associated with seven viruses viz., apricot pseudo chlorotic leaf spot virus (ApPCLSV), apricot latent virus (ApLV), ASGV, ASPV, ACLSV and peach chlorotic mottle virus (PCMV) [23]. Other researchers also reported the presence of known, divergent and novel viruses/viroids using next-generation sequencing in apples from China, Korea and Japan [11,15,24,25]. AHVd has been reported from apple trees exhibiting limb cracking and swelling; however, such symptoms were absent in AHVd-associated apple cultivars in our study, which may be due to the dominant role of other viruses (ApMV, ApNMV, ASGV and ASPV) in symptomatic trees. In one of our previous studies investigating the infection of AHVd in various cultivars based on RT-PCR, we found four out of six commercially grown cultivars were infected with this viroid [26]. However, further work is needed before this recently described and apparently widespread viroid can be definitively associated with a disease.

The genomes of ApNMV and ApMV consist of three RNA molecules (RNA1, RNA2, RNA3) of which RNA1 and RNA 2 encode the RNA-dependent RNA polymerase. The RNA1 encodes a protein containing conserved helicase and methyltransferase domains, whereas RNA2 encodes the conserved RNA polymerase domain. RNA 3 encodes for the movement protein and coat protein. This genome organization is similar to the viruses in the genus *Ilarvirus* [27]. Similarly, the retrieved genomes of ASGV and ASPV showed single ORF similar to the earlier-reported genomes of these viruses [28,29]. The genome of AHVd variants ranged in size from 434 nt to 440 nt, with clear distinct nucleotides at specific positions as indicated from the secondary structure. The nucleotide variability observed and phylogenetic analysis among the two isolates confirmed intra-isolate variability as observed by other workers [30,31]. The clustering of the identified viruses and viroids with viruses from countries such as Japan, Italy, China and the USA indicates the possible introduction of these viruses from these countries to India via the exchange of planting material either through cultivars or rootstocks. India is continuously importing exotic apple cultivars and rootstocks, both spur and standard types, from the Netherlands, Italy, Japan and the United States of America (https://seednet.gov.in, accessed on 19 August 2021), hence the introduction of viruses cannot be ruled out. The clonal propagation of apples and the free trade of planting material have spread viruses and viroids globally [19]. The presence of recombination events in different viruses identified using HTS has led to the presence of genetic variation among the viruses. Recombination and mutations are major players in plant virus evolution, the appearance of new strains/variants and adaptation to diverse hosts [32]. In the present study, the viruses present were of mixed infection in both varieties, which offers scope for recombination and evolution. The presence of mixed infection of more than one virus in the plants will make genetic exchanges between them possible, and even the formation of new hybrid viral species containing parts from the genomes of the distinct viral species, through recombination or reassortments, which eventually leads to speciation events, thus contributing to the expansion of the global Virome [17].

Based on the symptoms, an earlier incitant of AMD was named ApMV [12,13] and was also considered as the only cause, but through subsequent studies using RNAseq and RT-PCR, the association of novel viruses such as ApNMV has been confirmed from countries such as China, Korea and Japan [5,17]. In the present study, the two symptomatic plants (OS and GD) tested positive for ApNMV infection while ApMV was detected in only one symptomatic apple cultivar, GD, in mixed infection with ApNMV using RNA sequencing, whereas the asymptomatic cultivars, both ApMV and ApNMV, were absent and two latent viruses (ACLSV and ASPV) were present. In host plants, establishing the relation between symptom expression and specific viral infection can be further complicated by the presence of mixed infections of viruses/viroids [33,34,35,36]. During the present study, the mixed infection of viruses (ASPV, ASGV) and one viroid (AHVd) was also observed, apart from ApNMV and ApMV. However, in most perennial crops viz., apple, pear, grape and some stone fruits, ASPV and ASGV are present in mixed infections and are known to be latent in nature, i.e., usually symptomless in most commercially grown apple cultivars, hence could not be associated with mosaic disease [19,34,35]. AHVd, which is associated with diverse symptoms ranging from dieback to trunk splitting, was not found to show any of these symptoms in mosaic-infected samples. The absence of ApMV and ApNMV from asymptomatic cultivars in RT-PCR and the presence of other latent viruses (ASPV) provide a clear indication of the relationship between ApNMV or ApMV and apple mosaic disease. The presence of ApNMV in two mosaic-infected cultivars and ApMV in only one cultivar indicates that ApNMV could be a major incitant of the mosaic disease in commercially grown apple cultivars in India. In our previous studies, we reported the association of ApNMV as a more likely cause than ApMV by conducting the survey among various apple cultivars exhibiting either mosaic or mosaic–necrotic symptoms in major apple-growing districts of Kashmir valley. ApNMV was found to be associated with five cultivars of apple (Red Delicious, Golden Delicious, Oregon Spur, Royal Delicious and Fuji Aztec) whereas ApMV was found to be associated with only two cultivars (Red Delicious and Golden Delicious) [37]. Despite the presence of ApNMV in the GD cultivar, necrosis was absent, which may be due to the presence of a higher percentage of ApMV reads than ApNMV. We also assume that ApNMV was not present in the optimum titer to cause necrosis in the GD cultivar. Nevertheless, it is very difficult to specify whether the necrotic symptoms are caused by ApNMV as it was found to be associated with both types of symptoms (mosaic and necrosis). The symptoms of either mosaic or necrosis development can be proven only by developing infectious clones of both the viruses (ApMV and ApNMV) followed by inoculation on apple seedlings either individually or in combination.

## 4. Materials and Methods

### 4.1. Plant Source

During spring of 2019, leaves showing mosaic/necrotic mosaic were collected from 10–12-year-old trees of the apple cultivars OS, GD and RF grown in the field gene bank of ICAR-Central Institute of Temperate Horticulture, Srinagar, J&K that were either symptomatic or asymptomatic (RF). Pooled leaf samples from one tree of each cultivar were subjected to virome analysis by sequencing the total RNA (RNA-Sequencing) using Hiseq Illumina 2500sequencer at Nucleome Biotech, Hyderabad, India.

### 4.2. RNA Isolation, Library Preparation and Illumina HiSeq Sequencing

Total RNA of the two samples was extracted using the QIAGEN RNeasy^®®^ Plant minikit (Qiagen Germany) as per the manufacturer’s protocol and instructions. The samples were quantified and checked qualitatively by NanoDrop (Thermo Fisher Scientific, Mumbai, India), the Agilent 2100 Bioanalyzer (Agilent Technologies, Palo Alto, CA, USA) and agarose gel (1%) electrophoresis. One microgram of total RNA with an RNA integrity number (RIN) value of >7 was used for library preparation. Ribo-zero treatment was performed on the total isolated RNA using the Ribo-Zero Magnetic Kit (Epicentre, Madison, WI, USA) to remove ribosomal RNA (rRNA). A random-primed cDNA library was constructed using a TrueqRNAB Sample PrepKit (Illumina, San Diego, CA, USA) The NGS library preparations were prepared according to the manufacturer’s instructions and protocol (NEBNext^®®^ UltraTM RNA Library Prep Kit for Illumina^®®^, New England Biolabs, Massachusetts USA). The libraries were sequenced on an Illumina HiSeq 2500 sequencer (Biomarker Technologies Co., Ltd., Rohnert Park, CA 94928, USA.).

### 4.3. Sequence Processing and De Novo Assembly

The raw read data in fasta format were initially processed using in-house perl scripts to obtain clean reads by removing adapters and multiple-N-containing and low-quality reads. The trimming and cleaning of reads were performed by removing sequences smaller than 120 nucleotides. For clean data, the GC-content, phred quality scores (Q20 and Q30) and sequence duplication level were calculated. To eliminate the low-quality sequences (phred score ≤ 25) and short reads, paired-end reads were filtered by the QC Toolkit v2.3.3. The high-quality clean data were used for all downstream analyses. The sequence data in the form of raw reads obtained from OS and GD were run through a taxonomic and classification tool, Kaiju version 1.6.3 using the “NCBI BLAST nr + euk” database as a reference to identify the sequences of non-host origin. Trinity version 2.3.0 and Velvet version 1.1 [38] with k-mer = 17 were used for de novo assembly of reads into contigs [39]. For comparative analysis, the relative numbers of individual viruses/viroids in each sample and the percentage distribution of reads pertaining to viruses/viroids were calculated by dividing the number of virus-/viroid-associated reads by the total number of clean reads (×100). Similarly, the individual virus- and viroid-associated read percentages were calculated by dividing the number of individual virus- or viroid-associated reads by the total number of virus/viroids reads (×100) [17].

### 4.4. Virus Identification and Genome Reconstruction from Assembled De Novo Contigs

Virus-/viroid-related contigs were filtered using SAM tools version 1.9 [40]. The assembled contigs were further annotated by BLASTn and BLASTx analysis searches in the virus/viroid reference database in the NCBI nucleotide GenBank. The consensus sequences for particular viruses/viroids, obtained after de novo assembly, were considered as the complete/near-complete genome. The open reading frames (ORFs) were found in assembled viral genomes using the ORF finder from NCBI (https://www.ncbi.nlm.nih.gov/orffinder, accessed on 28 June 2021).

### 4.5. Phylogenetic and Recombination Analyses of Identified Viruses/Viroids

Complete genome sequences of viruses/viroids retrieved from NGS data were submitted to GenBank. In order to find the phylogenetic relationship among our isolates, the complete genome sequences were retrieved for viruses homologous to our isolates from the NCBI database for viruses/viroids (www.ncbi.nlm.nih.gov, accessed on 18 May 2021). The Clustal W multiple-alignment program was used for the alignment of genome sequences, and the sequence identity matrix was calculated using BioEdit (7.2) with the default setting. The phylogenetic trees of all viruses/viroids were constructed using the neighbor-joining method (NJM) in MEGA.7.0, with confidence levels for the phylogroups estimated using 1000 bootstrap replicates [41]. The Recombination Detection Program RDP4 Version 4 was used to determine the recombination events. The number of recombination events, recombination breakpoints and major and minor parents of recombinants were analyzed using various algorithms viz., RDP, Chimaera GENECONV, SISCAN, BOOTSCAN, 3Seq and Maxchi in the RDP4 software package. The settings with default parameters were used, and recombination events detected by more than 4 algorithms were considered for analysis. The single-nucleotide variations were identified using the SAM tools program. The single-nucleotide variants (SNV) were identified by extracting alignments using BLASTn followed by pair wise alignment, and the spurious ones were filtered due to insufficient coverage and subsequently removed.

### 4.6. Secondary Structure Determination in Viroid

The sequences obtained from NGS data were further used to predict the secondary structure the viroid using RNA structure software Version 6.2 [42].

### 4.7. Confirmation of Virus and Viroid with RT-PCR and Sanger Sequencing

For the confirmation of viruses/viroids, total RNA was isolated using the Spectrum™ Plant Total RNA Kit (Sigma Aldrich Co, Gillingham, United Kingdom) as per the manufacturer’s protocol, from symptomatic as well as asymptomatic leaf samples. The RNA was checked qualitatively using NanoDrop (Thermo Fisher Scientific Inc.). The first strand of c-DNA was synthesized using the FIRE Script^®®^ RT cDNA synthesis kit (Teaduspargi 11 Tartu Estonia) as per the manufacturer’s protocol, with a slight modification of the reverse transcription temperature, which was maintained for 25 min. The reaction mixture of 20 μL containing the reverse primer of the specific virus was incubated in PCR at 25 °C for 8 min, 50 °C for 30 min and 85 °C for 5 min. Later, the cDNA was used in PCR amplification. For RT-PCR, specific primer pairs (Table S1) reported earlier was used to amplify genomic regions corresponding to the coat protein or replicase genes. RT-PCR was performed in a 30-cycle program with denaturation at 94 °C for 30 s, annealing at 50 °C for ApNMV, 53 °C for ApMV [35], 50 °C for ASGV and 55 °C for ASPV and AHVd for 45 s [31,37], followed by extension at 72 °C for 30 s with a final extension at 72 °C for 7 min. The reagents were the same except the cDNA template and forward and reverse primers of specific viruses/viroids. The amplicon of viruses was electrophoresed in 0.8% agarose gel in 0.5X TAE buffer (pH 8.0) at 85V, and a 1kb DNA ladder was used to estimate the amplicon size (New England Biolabs starting from 500bp and Thermo scientific starting from 250bp). The amplicons were visualized under UV light in a gel-documentation system (Bio Rad, Gel Doc XR system 170–8170). The DNA fragments were eluted in the gel using an elution gel extraction kit as per instructions from the manufacturer (Promega Wizard^®®^ SV Gel and PCR Clean-Up System, Madison, USA). The purified eluted DNA products were cloned in pGEM-T cloning vector (Promega, Madison, USA), and for multiplication, a competent DH5 α bacterial cell was transformed with a recombinant plasmid. Blue-white selection, restriction digestion and colony PCR were used to confirm the positive clones. The sequencing was performed for positive clones from all the viruses in both directions by Eurofins genomics Bengaluru, Karnataka, India. The nucleotide sequences were analyzed using the basic local alignment search tool (BLASTn) (http://www.ncbi.nlm.nih.gov, accessed on 15 February 2022). The leaf sample from the asymptomatic cultivar Red Fuji was taken as a negative control in the PCR reaction.

## 5. Conclusions

Fruit trees, including apples that are perennial in nature and propagated vegetatively are considered storehouses of viruses/viroids. The genetic diversity of the viruses/viroids co-infecting apple trees implies that synergisms among them may play a significant role in determining the phenotype of diseased plants. In the present study, RNAseq detected the viruses/viroids associated with mosaic symptoms. The indistinguishable nature of symptoms induced by ApMV or ApNMV or the presence of latent infection of other viruses means accurate and robust diagnostics is indispensable in the early stages of nursery establishment to stop the dissemination of viruses via rootstocks and scion wood. Hence HTS-based approaches will enable plant virologists to identify and characterize known viruses along with novel and divergent viruses/viroids for healthy orchard establishment. Our results from this study clearly show the presence of a mixed infection of different viruses in apple plants along with genetic diversity. This study will expand the nature and range of viruses/viroids infecting apple trees and will also provide an overview of pathogenic agents associated with AMD. It is likely to help in developing long-term and sustainable viral disease management strategies in apples in India.

## Figures and Tables

**Table 1:** Read statistics and percentage of virus and viroid reads via next-generation sequencing Illumina Hiseq 2500 from three apple cultivars.

Cultivar	Total No. of Raw Reads (Million)	Total No. of Clean Reads (Million)	Total No. of Viral Reads	Total No. of Viroidal Reads	Percentage of Viral Reads	Percentage of Viroidal Reads
Oregon Spur (S)	41.41	40.79	558314	349452	1.36	0.85
Golden Delicious (S)	32.58	32.35	57381	102943	0.18	0.31
Red Fuji (AS)	27.39	27.15	14312	12	0.05	0

AS: Asymptomatic, S: Symptomatic.

**Table 2:** Percentage distribution of individual viral reads, number of contigs obtained from each virus and total sequence length retrieved from two cultivars of Oregon Spur and Golden Delicious after RNA-Seq.

Name of Virus	Cultivar Oregon Spur			Cultivar Golden Delicious			Cultivar Red Fuji		
	Percentage (%) Distribution of Reads	Number of Contigs Assembled	Total Sequence Length (bp) Retrieved	Percentage (%) Distribution of Reads	Number of Contigs Assembled	Total Sequence Length (bp) Retrieved	Percentage (%) Distribution of Reads	Number of Contigs Assembled	Total Sequence Length (bp) Retrieved
ApNMV	59.9	10	8258	32.17	8	5432	0	0	0
APMV	0.00	0	0	63.2	12	10,034	0	0	0
ASGV	12.84	8	7561	1.2	7	6554	0	0	0
ASPV	2.36	32	62,060	0.0	0	0	1.45	29	8000
AHVd	77.5	3	440	83.1	17	434	0	0	0
ACLSV	0	0	0	0	0	0	2.49	15	11,474

**Table 3:** The genome size, organization, sequence similarity and accession numbers of viruses identified from apple leaf samples from next-generation sequencing.

Virus Name	RNA	Genome Size (kb)	Sequence Similarity (%)	Reference Accession	UTR-5′	ORF1	ORF2	ORF3	ORF 4	ORF5	UTR-3′	Accession No. Received
ApMV-GD *	1	3.274	99.3	KY965059	1−76	77–3220 (RdRp)					3221–3274	MN822137
	2	2.982	94.30	HE574163	1−80	81–2714 (RdRp)					2715–2982	MN822138
	3	1.991	99.7	KY971019	1−155	156–1016 (MP)	1111–1782 (CP)				1783–1991	MN822139
ApNMV-OS *	1	3.386	88.25	MG924895	1−58	59–3229 (RdRp)					3230–3386	MN832844
	2	2.782	84.5	KY808383	1−54	55–2622 (RdRp)					2623–2782	MT019877
	3	1.969	96.31	KY808378	1−208	209–1051 (MP)	1155–1820 (CP)				1821–1969	MN832845
ASGV-OS *	1	6.487	90.23	JN701424.1	1−27	28–6354 (RdRp)	4779–5741 (MP&CP)				6355–6487	MN786531
ASPV-GD *	1	9.206	84.38	MK923754	1−50	51–6608 (RdRp)	6720–7391 (TGB1)	7392–7755 (TGB2)	7664–7891 (TGB3)	7965–9155 (CP)	9156–9206	MN887352
ACLSV-RF *	1	7.552	89.0	D14996	1−151	152–5809 (RdRp)	5721–7100 (MP)	6616–7365 (CP)			7366–7547	MN872427
AHVd-OS *	1	440bp	99.00	MH649334	-	-	-	-	-	-	-	MN786530
AHVd-GD *	1	434bp	93.00	MH643720	-	-	-	-	-	-	-	MN786529

(*) The OS and GD are prefixed to virus because the whole genome was retrieved from that cultivar.

**Table 4:** The recombination events detected along with major and minor parents and *p*-value of viruses detected in apple trees using next-generation sequencing.

Virus	Event	Recombinant	Minor Parent	Major Parent	Break Point Start	Break Point End	Method	*p*-Value
ApMV (RNA 2)	A1	MN822138	KY965082	KY883319	Undetermined	2539	**G**BMC	1.77 × 10^−14^
	A2	MN822138	KY965082	KY883319	2836	Undetermined	GBM**C**	5.22 × 10^−3^
ApNMV (RNA 3)	A1	MN832845	Unknown	KY808378	Undetermined	Undetermined	**G**BMCS	1.85 × 10^−4^
ASPV	A1	MN887352	KY702580	MK923754	5117	Undetermined	BM**C**S	1.64 × 10^−3^
	A2	MK923754	**MN887352**	LC475150	Undetermined	6728	BM**C**S	1.264 × 10^−4^
ASGV	A1	MN786531	Unknown	JN701424	5876	Undetermined	GB**M**CS	1.98 × 10^−6^
	A2	MN786531	KR185346	KX668488	Undetermined	Undetermined	GB**M**CS	2.98 × 10^−2^
ACLSV	A1	D14996	Unknown	**MN872427**	6503	7342	GB**M**CS	1.427 × 10^−9^
	A2	MK929793	**MN872427**	Unknown	6843	7337	GBM**C**S	2.922 × 10^−6^

Only events supported by at least four of the different RDP4-implemented methods are reported in the table. G, GENECONV; B, Bootscan; M, MaxChi; S, SiScan; C, Chimaera; 3, 3Seq. The lowest significant *p*-value is indicated in bold font.

## Data Availability

All the data is available with corresponding author which will be made available on request.

## Supplementary Materials

The following supporting information can be downloaded at: https://www.mdpi.com/article/10.3390/plants11050675/s1, Table S1: List of primers, their target region and product size.; Figure S1: Open Reading frames identified using NCBI ORF finder in genomes of viruses.; Figure S2: Recombination events in reconstructed viral genomes using RDP4 program.
