# Supplementary material for: High-Throughput RNA Sequencing of Mosaic Infected and Non-Infected Apple (Malus × domestica Borkh.) Cultivars: From Detection to the Reconstruction of Whole Genome of Viruses and Viroid
Source: Plants (Basel). 2022 Mar 1;11(5):675. doi: 10.3390/plants11050675 (PMC8912866; doi:10.3390/plants11050675)
Supplement: Supplementary file 1 [file plants-11-00675-s001.zip › plants-1502723-supplementary.pdf]

plants MDPI

Table S1. List of primers, their target region and product size.

Target	Primer Name	Primer sequence	Product size (bp)
CP	ApMV	F5'CTCAAGCGAACCCGAATAAGGGTAAGAA-3'<br>R3'TCGTCGATAAGTAGAACATTCGTCGGTATTGTC-5'	550
Rep-gene	ASGV	F 5'CATATGTTCACTGAGGCAAAAGCTG-3'<br>R 3´GGATCCAGAAAACCCATCAAAGACTT5´	230
CP	ASPV	F5'ATGTCTGGAACCTCATGCTGCAA-3'<br>R 3'TTGGGATCAACTTTACTAAAAGCATAA-5'	370
CP	ApNMV	F 5´ CTTGCGTGCAATCGATATGG-3'<br>R 3´TCATCTCAACCTAGACATCC-5'	670
Full genome	AHVd	F 5'CCTTCCTGATGAGTCCGTTCCA-3'<br>R3'CTAATAGCCTCCGACCGTCAT-5'	440
RNA 2	ApNMV	F 5´ ACCGAGTTTATGTTTACCA-3'<br>R 3´CCAAAGGGATTGCCCCGTA-5'	700

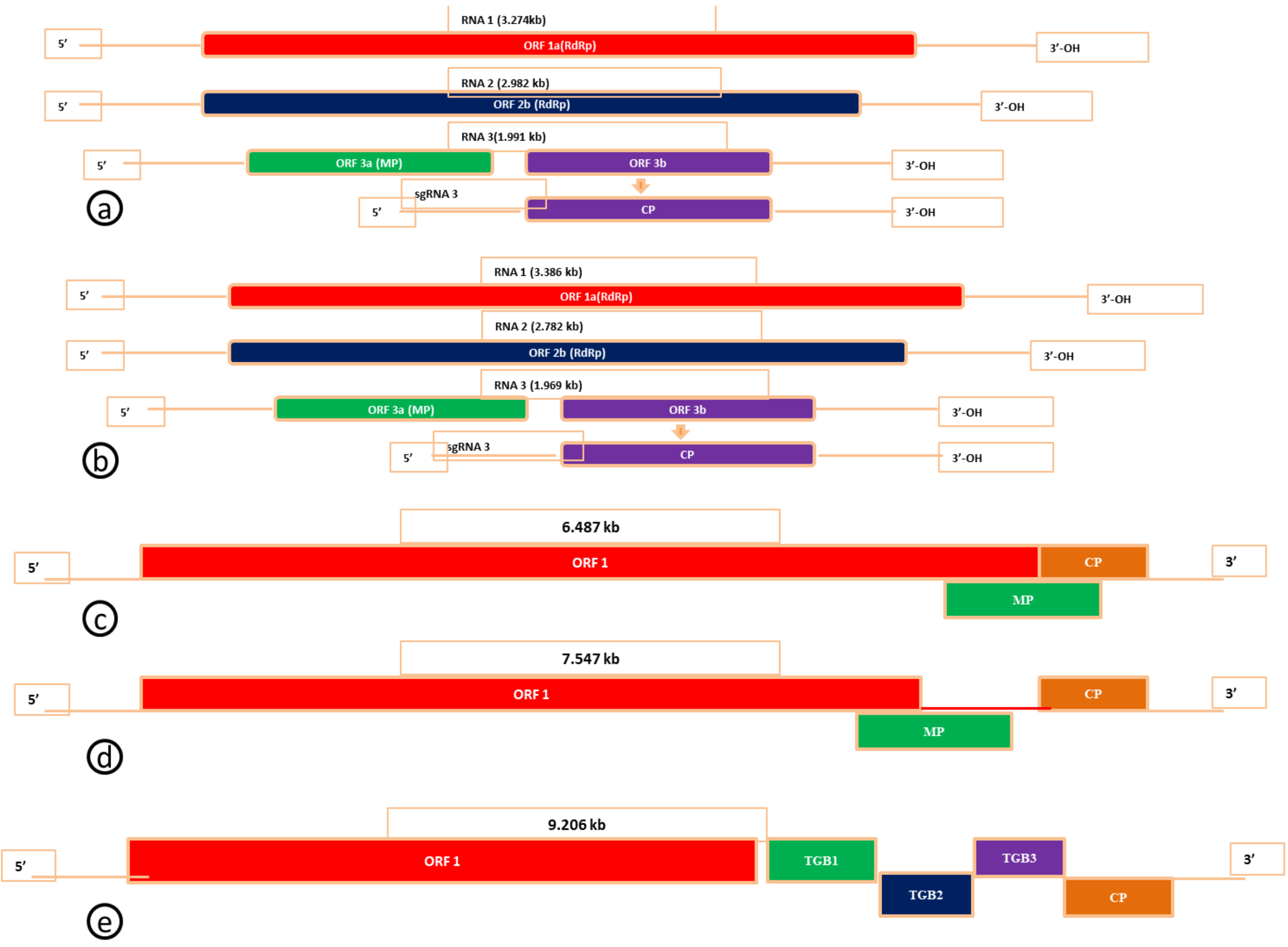

Figure S1. Open Reading frames identified using NCBI ORF finder in genomes of viruses (**a**-ApMV, **b**-ApNMV, **c**-ASPV, **d**-ASGV, **e**-AHVd) detected using RNA-Seq.

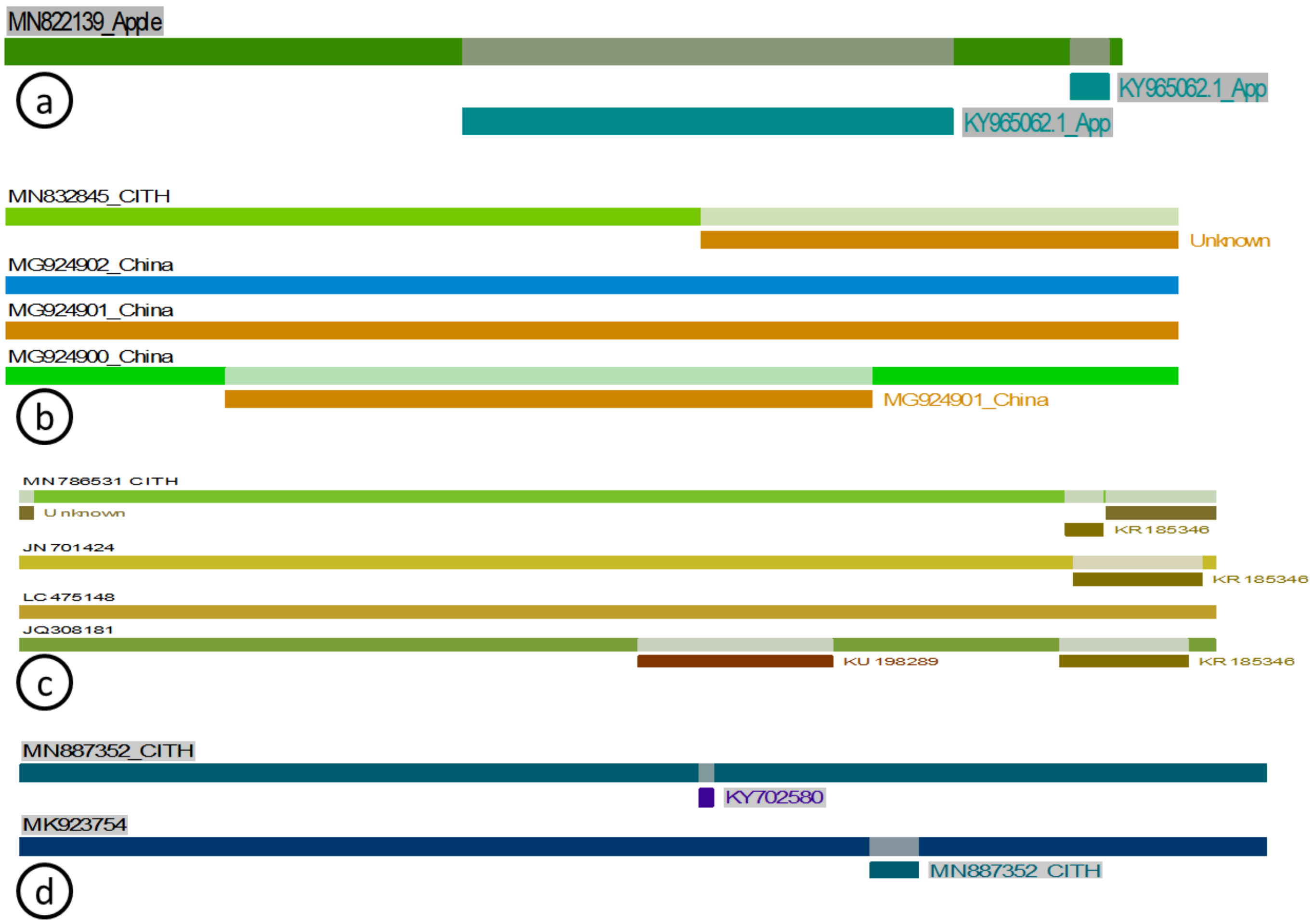

Figure S2. Recombination events in reconstructed viral genomes using RDP4 program. The positions of identified recombination events in our isolates are indicated in a different color in the genome, **a**- ApMV, **b**-ApNMV, **c**-ASPV, **d**-ASGV.
